# Photodetection of early human bladder cancer based on the fluorescence of 5-aminolaevulinic acid hexylester-induced protoporphyrin IX: a pilot study

**DOI:** 10.1038/sj.bjc.6690338

**Published:** 1999-04

**Authors:** N Lange, P Jichlinski, M Zellweger, M Forrer, A Marti, L Guillou, P Kucera, G Wagnières, H van den Bergh

**Affiliations:** Institute of Environmental Engineering, Swiss Federal Institute of Technology (EPFL), CH-1015 Lausanne, Switzerland; Department of Urology, CHUV Hospital, CH-1011 Lausanne, Switzerland; Institute of Physiology, University of Lausanne, CH-1005 Lausanne, Switzerland; Department of Pathology, CHUV Hospital, CH-1011 Lausanne, Switzerland

**Keywords:** 5-aminolaevulinic acid, 5-aminolaevulinic acid hexylester, photodynamic therapy, fluorescence, protoporphyrin IX, human bladder cancer

## Abstract

Exogenous administration of 5-aminolaevulinic acid (ALA) is becoming widely used to enhance the endogenous synthesis of protoporphyrin IX (PpIX) in photodynamic therapy (PDT) and fluorescence photodetection (PD). Recently, results have shown that the chemical modification of ALA into its more lipophilic esters circumvents limitations of ALA-induced PpIX like shallow penetration depth into deep tissue layers and inhomogeneous biodistribution and enhances the total PpIX formation. The present clinical pilot study assesses the feasibility and the advantages of a topical ALA ester-based fluorescence photodetection in the human bladder. In this preliminary study 5-aminolaevulinic acid hexylester (h-ALA) solutions, containing concentrations ranging from 4 to 16 mM, were applied intravesically to 25 patients. Effects of time and drug dose on the resulting PpIX fluorescence level were determined in vivo with an optical fibre-based spectrofluorometer. Neither local nor systemic side-effects were observed for the applied conditions. All conditions used yielded a preferential PpIX accumulation in the neoplastic tissue. Our clinical investigations indicate that with h-ALA a twofold increase of PpIX fluorescence intensity can be observed using 20-fold lower concentrations as compared to ALA. © 1999 Cancer Research Campaign


*British Journal of Cancer* (1999) **80**(1/2), 185–193
© 1999 Cancer Research Campaign
Article no. bjoc.1998.0338

### Photodetection of early human bladder cancer based on the fluorescence of 5-aminolaevulinic acid hexylester-induced protoporphyrin IX: a pilot study

**N Lange[1], P Jichlinski[2], M Zellweger[1], M Forrer[1], A Marti[2], L Guillou[4], P Kucera[3], G Wagnières[1] and H van den Bergh[1]**

[1]Institute of Environmental Engineering, Swiss Federal Institute of Technology (EPFL), CH-1015 Lausanne, Switzerland; [2]Department of Urology, CHUV Hospital, CH-1011 Lausanne, Switzerland; [3]Institute of Physiology, University of Lausanne, CH-1005 Lausanne, Switzerland; [4]Department of Pathology, CHUV Hospital, CH-1011 Lausanne, Switzerland

**Summary** Exogenous administration of 5-aminolaevulinic acid (ALA) is becoming widely used to enhance the endogenous synthesis of protoporphyrin IX (PpIX) in photodynamic therapy (PDT) and fluorescence photodetection (PD). Recently, results have shown that the chemical modification of ALA into its more lipophilic esters circumvents limitations of ALA-induced PpIX like shallow penetration depth into deep tissue layers and inhomogeneous biodistribution and enhances the total PpIX formation. The present clinical pilot study assesses the feasibility and the advantages of a topical ALA ester-based fluorescence photodetection in the human bladder. In this preliminary study 5-aminolaevulinic acid hexylester (h-ALA) solutions, containing concentrations ranging from 4 to 16 mM, were applied intravesically to 25 patients. Effects of time and drug dose on the resulting PpIX fluorescence level were determined in vivo with an optical fibre-based spectrofluorometer. Neither local nor systemic side-effects were observed for the applied conditions. All conditions used yielded a preferential PpIX accumulation in the neoplastic tissue. Our clinical investigations indicate that with h-ALA a twofold increase of PpIX fluorescence intensity can be observed using 20-fold lower concentrations as compared to ALA.

**Keywords:** 5-aminolaevulinic acid; 5-aminolaevulinic acid hexylester; photodynamic therapy; fluorescence; protoporphyrin IX; human bladder cancer

Fluorescence photodetection (PD) and photodynamic therapy (PDT) are techniques currently under clinical assessment for both visualization and local destruction of malignant tumours and premalignant lesions. One drawback of these methods found with some photosensitizers is a more or less long-term cutaneous photosensitivity (Wagnières et al, 1998; Dougherty et al, 1990). A more recent strategy for administering photosensitizers involves the application of 5-aminolaevulinic acid (ALA) in order to stimulate the formation of protoporphyrin IX (PpIX) in situ. The exogenous ALA bypasses the negative feedback control from haem to ALA synthase that catalyses the condensation of glycine and succinyl–coenzyme A (CoA). Given in excess, exogenous ALA thus can result in a temporary accumulation of PpIX, in particular, in cells with higher metabolic turnover. Since PPIX has fairly good photosensitizing properties (Cox et al, 1982; Kennedy et al, 1990) proposed ALA as a possible photodynamic agent. Following this pioneering work, this treatment modality has been widely studied for various cancers (Kennedy et al, 1992; Peng et al, 1992; Svanberg et al, 1994).

As well as for the PDT of malignant or premalignant lesions, ALA-induced PpIX is now being used for the detection of such lesions. This technique has been shown to work, among other applications, in urology, where easy instillation in the bladder, combined with the fact that this organ is readily accessible endoscopically, makes it an ideal object. Alongside classical techniques such as cytology or white light examination, fluorescence PD by ALA-induced PpIX provides some advantages (Leveckis et al, 1994; Kriegmair et al, 1996; Jichlinski et al, 1997). This inspection modality allows an exact mapping which pinpoints, with a high level of sensitivity and specificity, the locations of carcinoma in situ (CIS) as well as early stages of cancer-like dysplasias, which are normally difficult to recognize under white light examination.

However, when using topically instilled ALA for the PDT of CIS and precancerous lesions, this modality appears to be limited by the amount of ALA that enter the target cells or by the tissue penetration and the distribution of the resulting PpIX in the targeted tissue. Almost all of these possible disadvantages accompanying the use of ALA can be ascribed to the physical–chemical properties of the molecule itself. Applied under physiological conditions, ALA is a zwitterion (Novo et al, 1996). Because the lipid bilayer of biological membranes is relatively impermeable to charged molecules, the cellular uptake of ALA is shallow. Consequently, in order to increase the transport across cellular membranes, fairly high drug doses and increased administration times have to be used. This deficiency results in a low penetration depth (Warloe et al, 1992; Loh et al, 1993; Peng et al, 1995) and an ALA-induced PpIX distribution, which is not optimized for the PDT of the deep layers of nodular lesions in the urothelium (Iinuma et al, 1995; Chang et al, 1996) after topical ALA application.

Systematic studies have shown that the modification of a drug to an ester, an amide or a urethane by the addition of a long-chain hydrocarbon improves penetration through biological barriers (Bridges et al, 1979; Jain, 1987*a*, 1987*b*). After penetration into the cell, the ester derivative can then, for example, be hydrolysed back to the free ALA by non-specific esterases. Recently,

*Received 24 July 1998*
*Revised 8 October 1998*
*Accepted 15 October 1998*

*Correspondence to:* N Lange promising results were obtained with different alkylesters of ALA in vivo and in vitro (Kloek et al, 1996; Peng et al, 1996; Gaullier et al, 1997; Marti et al, 1998). These groups demonstrated that the application of esterified ALA derivatives results in an up to 25-fold increase in PpIX fluorescence levels as compared to ALA.

This report covers initial clinical investigations with 5-aminolaevulinic acid hexylester hydrochloride (h-ALA)-induced fluorescence PD in the human bladder. Following our preclinical studies (Marti et al, 1999), we selected h-ALA from the multitude of possible ALA-alkylesters because it represents a good compromise between water–urine solubility and sufficient PpIX formation capacity at low doses. Furthermore, h-ALA has been shown to lead to a homogenous distribution of PpIX-related fluorescence over the entire urothelium in our pig bladder model (Marti et al, 1998). In addition, it can be synthesized simply from ALA and hexanol (Kloek et al, 1997). The goal of this clinical pilot study was to test h-ALA as a potential candidate for improving both the PD and PDT in the urinary bladder. Therefore, topical application of h-ALA should result in higher PpIX formation than is the case with the same amount of ALA. It should enable shorter times between instillation and examination and lower drug concentrations while retaining the outstanding selectivity of ALA. This work presents a preliminary optimization of h-ALA-induced PpIX in respect to the resulting fluorescence intensities. Both the influence of the concentration and instillation time of h-ALA solutions on the total amount of PpIX were determined in vivo by the use of an optical fibre-based spectrofluorometer.

### MATERIALS AND METHODS

### Patients

Twenty-five patients (seven women and 18 men, four cases of ordinary ALA and 21 cases of h-ALA) have been involved in this first study conducted since August 1997. The mean age was 70 years, covering an age range of between 44 and 85. Local ethical committee approval was granted for this study, and written consent was obtained in each case.

### Preparation and administration of ALA and h-ALA

ALA (99%) was purchased from Merck (Darmstadt, Germany). Other chemicals (thionyl chloride 99% and 1-hexanol 99.9%) used for the synthesis of h-ALA were ordered from Fluka Chemie AG (Buchs, Switzerland) and were used without further purification.

The synthesis described here is a slight modification of the methods reported recently (Takeya, 1992; Kloek et al, 1996). In brief, 3.5 ml of thionyl chloride were added drop by drop under stirring to an excess (~ 10 ml) of 1-hexanol cooled on ice in an

**Table 1** Experimental instillation conditions used in the first clinical trials with h-ALA and normalized fluorescence levels on papillary tumours (pTa G2) obtained by normalization to reference cuvette

| Patient no. | Concentration (mM) | Instillation time (h) | Resting time (h) | Fluorescence signal (r.u.) |
|---|---|---|---|---|
| 1 | 4 | 2 | – | 16.2 |
| 2 | 4 | 2 | – | 11.2 |
| 3 | 4 | 4 | – | 34.5 |
| 4 | 4 | 4 | – | 20.5 |
| 5[b] | 8 | 2 | – | 22.1 |
| | | | | 38.4 |
| 6 | 8 | 2 | – | 36.2 |
| 7 | 8 | 2 | – | 46.7 |
| 8 | 8 | 2 | – | – |
| 9[b] | 8 | 2 | 2 | 151.1 |
| | | | | 102.0 |
| 10 | 8 | 2 | 2 | 115.8 |
| 11 | 8 | 2 | 2 | 147.5 |
| 12 | 8 | 4 | – | 66.4 |
| 13 | 8 | 4 | – | 72.6 |
| 14 | 8 | 4 | – | 63.4 |
| 15 | 8 | 4 | – | 73.8 |
| 16 | 8 | 4 | – | 94.4 |
| 17 | 8 | 4 | – | 77.1 |
| 18 | 8 | 4 | 2 | 102.7 |
| 19 | 8 | 4 | 2 | 95.0 |
| 20 | 16 | 2 | – | 15.8 |
| 21 | 16 | 2 | – | 16.7 |
| 22 | 180[a] | 4 | 2 | 54.0 |
| 23 | 180[a] | 4 | 2 | 43.6 |
| 24 | 180[a] | 4 | 2 | 46.2 |
| 25 | 180[a] | 4 | 2 | 45.3 |

[a]Instillation of the 180 mM solution of ALA.[b]Patient with two papillary tumours.

© Cancer Research Campaign 1999

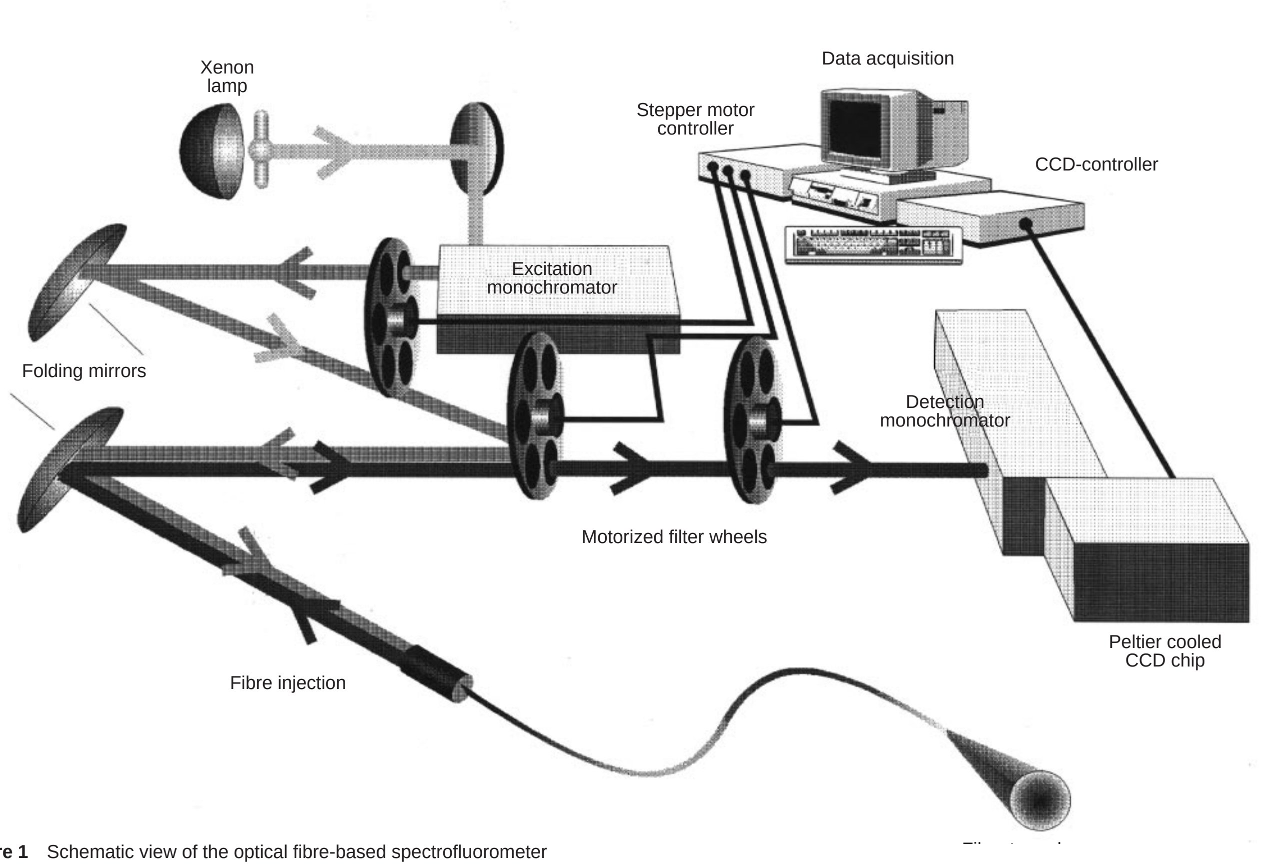

**Figure 1** Schematic view of the optical fibre-based spectrofluorometer argon atmosphere. The solution was stirred for a further 30 min to bring the reaction to completion; after warming up to room temperature, 2.5 g of ALA ($M_r$ = 167.6 g $mol^{-1}$) were added to the solution. The suspension was then stirred overnight at room temperature under argon. The final phase of the reaction was controlled on-line by thin layer chromatography (TLC) (TLC foils; Schleicher & Schuell, Merck, Darmstadt, Germany) in $CH_2Cl_2$/MeOH (9:1) stained by $KMnO_4$ (Rf = 0.6). Once the reaction was complete, the solvent and hexylchloride were removed under reduced pressure (~ 0.5 torr). The viscous residue was dissolved in warm methanol. Then a small amount of methanol was evaporated until the first crystals of the reaction product appeared. A small quantity of diethylether was added and h-ALA was allowed to crystallize on ice. This dissolving and recrystallizing procedure was then subsequently repeated until only one spot was recognized on the TLC, yielding 80–90% of h-ALA ($M_r$ = 251.8 g $mol^{-1}$) as a white powder. The product was characterized by proton nuclear magnetic resonance ($^1$H-NMR) with a 400 MHz (Bruker, Germany) spectrometer and identified as 5-aminolaevulinic acid hexylester hydrochloride. The purity (> 95%) was further verified by high performance liquid chromatography (HPLC) with UV/VIS detection at 270 and 350 nm respectively (data not shown). No other products were observed.

The ALA solutions were administered in accordance with standard protocol used in Lausanne's CHUV Hospital (Jichlinski et al, 1997). In brief, 1500 mg of ALA were dissolved in 38 ml of sterile water. Five millilitres of phosphate-buffered saline (PBS) were added and the pH was adjusted with a further 7 ml of aqueous sodium hydroxide (1N) to a value of pH 5.3. This solution with a concentration of 180 mM of ALA was sterilized by filtration through a Millipore filter (Millipore, Millex GS 0.22 µ) and stored at –18°C 1 day before measurements were conducted. The solution was instilled into patients' bladders using a 16 French Foley catheter 6 h prior to photodetection. Patients were asked to retain the solution for 4 h. Their bladders were evacuated 2 h prior to treatment.

Depending on the prodrug concentration to be applied, 50–200 mg (i.e. 4–16 mM) of crystalline h-ALA were dissolved in 35 ml of water. Then 13 ml of PBS were added to the aqueous solution and adjusted with 0.1 N hydrochloric acid to give the same pH value of 5.3. The solutions were instilled as described above. Table 1 summarizes the different conditions under which ALA and h-ALA were applied. All patients treated with ALA (four cases) and some instilled with h-ALA (five cases) had a supplementary resting time of 2 h after being exposed to the drug solution.

### Procedure

### *Bladder inspection under white light illumination*

Prior to further treatment or measurement, the actual status of the bladder was documented under white light illumination. The frame accumulation colour CCD camera (Storz, Tuttlingen, Germany), connected to a video recorder (JVC, Japan) and an RGB monitor (Sony, Japan) was plugged directly into the ocular of a 23.5 French cystoscope (Storz PDD, Tuttlingen, Germany) to record the standard endoscopic colour image.

*© Cancer Research Campaign 1999* 

*Fluorescence spectroscopy*

Fluorescence emission spectra were recorded with an optical fibre-based spectrofluorometer based on a Peltier-cooled CCD coupled to a spectrograph (Cromex 250, SI Instruments, Germany). The experimental setup is shown in Figure 1. Arranged on a trolley, the whole setup can be easily transported. Excitation light ($\lambda_{ex}$ = 405 nm) from a 75 W high-pressure Xenon lamp (UXL-75 XE, Ushio Inc., Japan) was spectrally resolved by a quarter meter monochromator (Chromex 250, SI Instruments, Germany) with a bandwidth of 5 nm and an excitation filter, SCHOTT BG3 (Schott AG, Mainz, Germany), mounted on a filter wheel. A stepper motor (SMC 100, Princeton Instruments Inc., USA) controlled this excitation filter wheel, which was equipped with different low-pass filters installed to purify the excitation light. Fully reflective mirrors and a dichroic mirror (Reynard DC 450; Reynard, USA), mounted on a second filter wheel, were used to feed the light into a 600 μm core silicone-clad silica fibre with perpendicular polished end-faces. Excitation energy measured at the distal end of the fibre tip was determined with a calibrated power-meter (Optical Power Meter 840, Newport, USA). Fluorescence emitted by any sample was collected with the same fibre and separated from the excitation light by the dichroic optics described above. A long-pass filter (Reynard FG 455) mounted on a third filter wheel made further spectral separation, virtually eliminating all reflected excitation light prior to acquisition. This filter setup allows the acquisition of fluorescence emission spectra between 450 and 900 nm. Detection based on this combination enables fast data acquisition combined with a low level of noise. The whole setup and data acquisition was controlled by a 486 personal computer using CSMA software (SI Instruments GmbH, Germany).

An aqueous solution of Rhodamine B ($c = 1 \times 10^{-6}$ mol $l^{-1}$) in a 10 mm quartz cuvette was used as a reference. Emission spectra of the reference were recorded before and after each measurement. All measurements were normalized to the peak value of the reference to give comparable results corrected for day-to-day fluctuations of the excitation light energy or detection pathway alignment.

After inspection of the bladder under white light, the distal end of the fibre was introduced via the biopsy channel of the cystoscope. A background measurement was performed in the centre of the bladder to allow the correction of the spectra for parasitic light and fluorescence generated by the fibre itself. Then the physician brought the distal end of the fibre directly into contact with the bladder wall.

*Bladder inspection under violet light illumination*

After measurement of the fluorescence spectra (see below) of healthy, cancerous and suspicious areas in the bladder, the camera was equipped with a long-pass filter (1 > 520 nm; Wratten filter No. 12, Kodak, Rochester, USA), positioned between the ocular of the cystoscope and the CCD-Chip. A footswitch allows the physician to place a bandpass filter (380–450 nm) in front of the 300 W Xenon arc lamp (Storz, Tuttlingen, Germany) to give about 150 mW of violet light at the end of the cystoscope. Excitation with violet light generated a visible pale-green autofluorescence of the healthy mucosa. As a result of the absorption of autofluorescence, the blood vessels of the lamina propria appear somewhat darker. Filtration of the light below 520 nm allows these sites to be distinguishable from zones containing high PpIX concentrations, appearing in a clear, bright, fluorescing red. To improve the fluorescence images, the camera was switched into frame accumulation mode for enhanced sensitivity. The integration times ranged from one-eighth to one-half of a second, depending on observation distance.

*Biopsy sampling and pathology*

Prior to transurethral resection of the bladder wall (TURB), a total number of 109 biopsies from fluorescent and non-fluorescent areas (average 5.2 per patient; guided by light-induced fluorescence after excitation at 405 nm) were taken from the patients treated with h-ALA solutions. Macroscopic fluorescence findings and locations were documented for each biopsy. All samples were sent for histopathological examination. The urothelial carcinomas were graded and staged according to the World Health Organization (WHO) 1973 classification (Mostofio et al, 1973) and the UICC/AJC 1992 system (Hermanek and Subin 1992) respectively. Flat intra-epithelial neoplastic lesions were graded according to the criteria of Nagy et al (1982) and classified as grade 1 (mild dysplasia), grade 2 (moderate dyslasia), grade 3 (marked dysplasia) and carcinoma in situ.

### RESULTS

### Macroscopic findings

All aqueous solutions of h-ALA stayed clear and colourless until use. Neither systemic nor local reactions following the examination with both h-ALA and ALA were observed under the conditions used in this study. Even the highest drug dose administered (16 mM) of h-ALA was well tolerated. h-ALA-induced synthesis of PpIX was observed in each patient. All papillary and planar tumours, also visible under white light cystoscopy, showed bright red fluorescence. This red fluorescence was found to demarcate the outline of the urothelial lesions with high precision. Using the violet light of the filtered Xenon arc lamp, it was possible to perform both fluorescence-guided biopsies as well as accurate resections of targeted tissues. Qualitatively, all conditions tested resulted in a clearly visible contrast between healthy and diseased sites of the bladder wall.

**Table 2** Correlation between histopathological finding and fluorescence diagnosis following h-ALA instillation

| Histopathological findings | Total number of biopsies | Fluorescence positive | Fluorescence negative |
|---|---|---|---|
| Healthy mucosa | 28 | 5 | 23 |
| Metaplasia | 1 | 1 | – |
| Hyperplasia | 3 | 3 | – |
| Dysplasia G1 | 12 | 10 | 2 |
| Dysplasia G2 | 5 | 3 | 2 |
| Dysplasia G3 | 2 | 2 | – |
| CIS | 11 | 9 | 2 |
| pTa G1 | 8 | 8 | – |
| pTa G2 | 14 | 14 | – |
| pTa G3 | 19 | 19 | – |
| pT1 G2–G3 | 4 | 4 | – |
| pT2a | 2 | 2 | – |
| Total | 109 | 80 | 29 |

 © Cancer Research Campaign 1999

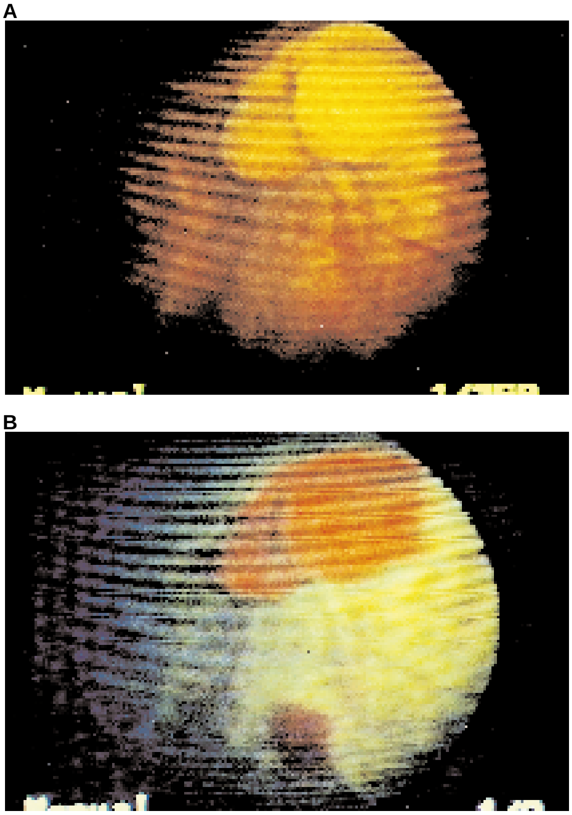

**Figure 2** Endoscopic view of a flat papillary tumour (pTa G2) after 2 h of h-ALA exposure (for description see text) under (**A**) white light, (**B**) violet light examination

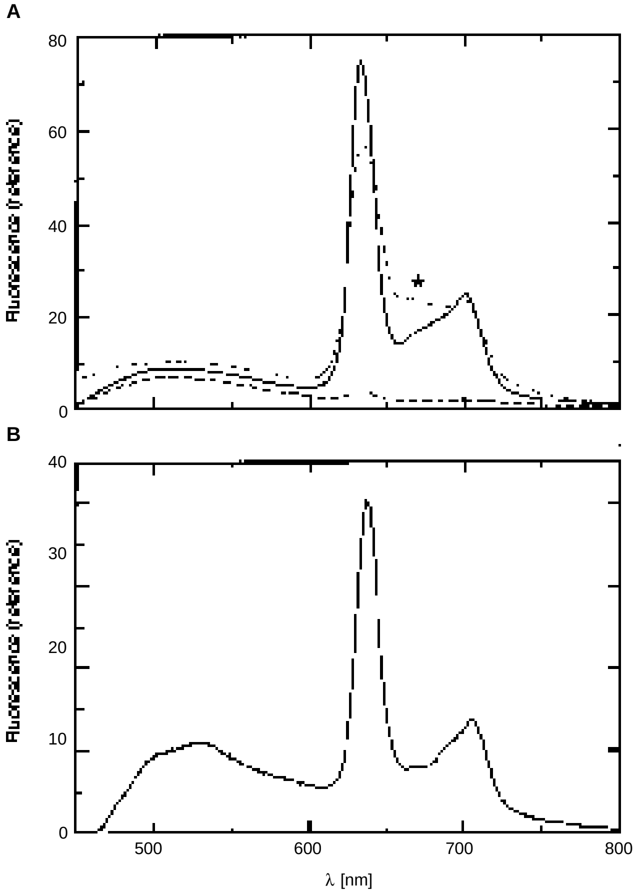

**Figure 3** (**A**) Fluorescence spectra of h-ALA-induced PpIX ($\lambda_{ex}$ = 405 nm) in normal mucosa (– – – –) and a papillary tumour (pTa G2, ——) after 4 h of instillation with 8 mM h-ALA (. . . .): a fluorescence peak (*) at 670 nm becomes visible due to photobleaching of PpIX. (**B**) Fluorescence spectra of ALA-induced PpIX ($\lambda_{ex}$ = 405 nm) in a papillary tumour (pTa G2, ——) after 6 h of installation with 180 mM ALA)

Figure 2 demonstrates the advantageous use of h-ALA- induced PpIX for the fluorescence diagnosis of human bladder cancer. The two pictures show a sequence of a white light (Figure 2A) and a violet light (Figure 2B) examination after instillation of 8 mM of h-ALA over a period of 2 h (patient no. 10). White light illumination shows two papillary tumours (pTa G2) situated below the air bubble of the bladder under investigation. Fluorescence PD of the same area (Figure 2B) indicates a further lesion [flat papillary tumour (pTa G2)] which is barely detectable under white light.

### Fluorescence findings and histopathological diagnosis

A total of 109 biopsies were taken under light-induced fluorescence from patients after instillation with h-ALA solutions. The correlation between the fluorescence findings and the histopathological analysis is summarized in Table 2. Thirty-two tissue samples were excised from healthy areas of the bladders investigated, containing eight samples, which were considered to be fluorescent. Histopathological diagnosis of the latter samples indicates the reasons for these ‘false positive’ responses. All these specimens showed tissular structures known for a higher cellular turnover, e.g. metaplasias, hyperplasias, chronic inflammation, or scar formation. In total, only six of the 77 biopsies taken from malignant and premalignant sites were not fluorescing. Three of these ‘false negative’ responses can be explained by nonoptimized conditions with regard to the concentrations of h-ALA applied (two moderate dysplasias; patient no. 4) as well as non-optimal incubation times (CIS; patient no. 1). A further CIS was missed (patient no. 13) probably due to an unusually long period of white light illumination preceding photodetection, resulting in the photobleaching of PpIX. Without exception, histopathologically-staged pta G1 or higher samples were found by fluorescence photodetection.

### Fluorescence spectroscopy

Fluorescence emission spectra were measured on a total number of 24 patients (20 h-ALA, four ALA) (Table 1). ALA- and h-ALA-induced porphyrins were excited at 405 nm on both healthy and malignant areas in the human bladder. The emission from the urothelial surface was spectrally resolved between 450 and 800 nm. A comparison of the emission spectra after ALA and h-ALA exposure is plotted in Figure 3. The total fluorescence intensity is normalized to the reference. As shown in this Figure, the characteristic emission bands of PpIX at λ = 635 nm and λ = 708 nm after excitation in the Soret Band are clearly visible. According to the spectral shape, the fluorescence is attributed to PpIX. In none of the spectra recorded in vivo, an indication of porphyrins other than PpIX could be found. Depending on the duration of white and violet light examinations before fluorescence

© Cancer Research Campaign 1999

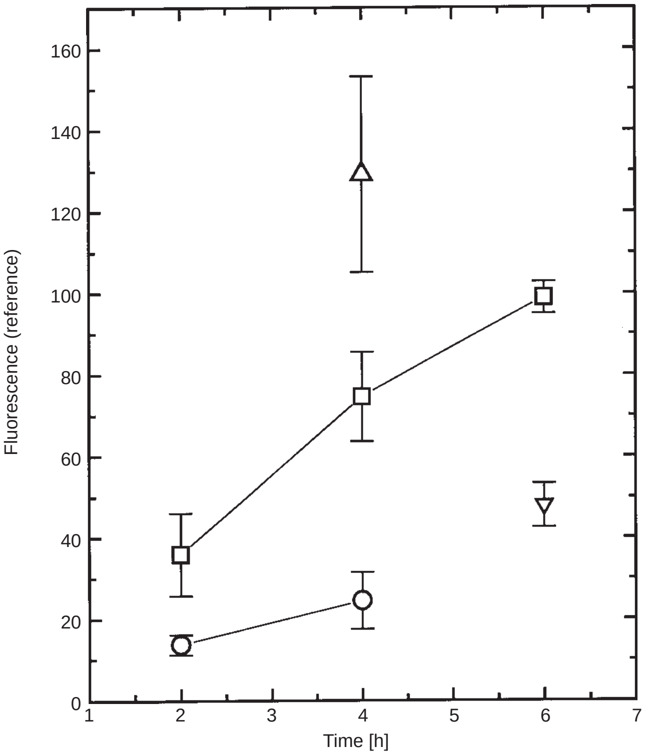

**Figure 4** Effect of instillation time on the relative PpIX fluorescence intensity at 636 nm in papillary tumours (pTa G2) (□: 8 mM h-ALA; ○: 4 mM h-ALA; △: 8 mM h-ALA (2 h of instillation + 2 h of resting time); ▽: 180 mM ALA)

measurements, a peak, attributed to a PpIX photobleaching product, of around 665–675 nm appeared (dotted line in Figure 3A). The appearance of this supplementary fluorescence peak results in a line broadening because of overlapping fluorescence emission peaks and may suggest some degree of heterogeneity. In addition to the fluorescence emission spectra recorded on a papillary tumour pTa G2 after 4 h of h-ALA exposure, the corresponding spectra obtained on a healthy area were plotted (dashed line in Figure 3A). From these spectra, it can be seen that the healthy mucosa's autofluorescence of around 513 nm exceeds the two typical fluorescence peaks of PpIX at 636 nm and 708 nm. All samples taken from sites with these fluorescence characteristics were confirmed as healthy after histopathological examination. Evaluation of all fluorescence data available reveals that papillary tumour had the highest emission intensities. Premalignant lesions, such as dysplasias and carcinoma in situ, generally showed lower PpIX fluorescence intensities compared to malignant lesions. However, no direct relationship has been discovered between histopathological grading and relative fluorescence values.

### Effect of exposure time and concentration

Three different solutions containing 50 mg (4 mM, four patients), 100 mg (8 mM, 15 patients) and 200 mg (16 mM, two patients) of h-ALA in 50 ml of the solvent were instilled in human bladders between 2 and 6 h prior to the fluorescence measurements (Table 1). Increased red fluorescence due to enhanced PpIX formation in pre-malignant and malignant lesions compared to the surrounding healthy sites was observed at all applied conditions. In order to quantify the resulting PpIX fluorescence, emission spectra were collected from different sites of the treated bladders. The fluorescence intensities of papillary tumours pTa graded G2 or G3 at 636 nm were chosen as standard in order to determine the influence of the different treatment conditions applied. This selection is based on the presence of this type of lesion in each bladder examined. Table 1 summarizes the influence of the different conditions on the relative fluorescence intensities of the PpIX emission band at 636 nm.

Analysis of the data available from patients exposed for 2 h (patient nos 1, 2, 5, 6, 7, 20 and 21) to different h-ALA concentrations indicates a strong concentration dependent on the PpIX fluorescence. It appears that, within 2 h, a solution of 8 mM h-ALA generates the highest fluorescence levels as compared to 4 mM and 16 mM of h-ALA. In Figure 4, the time course of the relative fluorescence intensity is plotted for h-ALA concentrations of 4 mM and 8 mM. An increase of fluorescence intensity with instillation time was observed in the two solutions. In addition, Figure 4 shows that, taking both the total fluorescence and the slope of the graphs into consideration, an instillation of 8 mM h-ALA solution is more efficient than that of a 4 mM solution.

In the course of our preliminary clinical study, a total of four patients (nos 8–11) were instilled under slightly different conditions. The patients' bladders were exposed to the solutions for 2 h. Following this exposure time, the bladders were emptied and the patients were allowed a supplementary resting time of 2 h. From Figure 4, it is clear that following this '(2+2)-concept' significantly enhanced fluorescence levels can be obtained compared to permanent exposure to the drug for 4 h.

The comparison of the relative fluorescence intensities of an 8 mM h-ALA solution and a 180 mM solution of ALA under similar conditions (4 h of instillation, 2 h of supplementary resting time) clearly demonstrates the advantage of using h-ALA. A treatment under these conditions with a topical 8 mM h-ALA solution resulted in a twofold increase of the fluorescence signal as compared to topical 180 mM ALA. After only 4 h of h-ALA exposure (8 mM), the relative fluorescence intensity already exceeded that induced by ALA (180 mM) 6 h after instillation.

### DISCUSSION

Bladder cancer is a fairly common disease, appearing between the ages of 50 and 70 (Richie et al, 1989; Levi, 1993). This cancer, characterized by a high incidence (Levi, 1993), can appear in many distinct morphological forms, single or multiple, visible such as papillary or invisible such as 'flat' atypical lesions, mainly represented by low- or high-grade dysplasia or CIS. Bladder tumour multiplicity and the presence of these different forms of atypia are indicators of poor disease prognosis. Recognition of all visible or invisible lesions is therefore a prerequisite for any kind of treatment, with the aim of reducing the risk of progression or the rate of recurrence.

Although topical application of ALA has proved to be a helpful and reliable tool in fluorescence photodetection of invisible lesions in human bladder disease (Kriegmair et al, 1994; Jichlinski et al, 1996), some problems remain due to ALA's poor bioavailability. A small hydrophilic amino acid like ALA does not penetrate into all tissue compartments with great ease. Hence its concentration in tissue may remain relatively low and its distribution somewhat heterogeneous. Consequently, high drug doses over long instillation periods have to be used.

 © Cancer Research Campaign 1999

Three different concepts have been proposed to enhance the ALA-induced PpIX formation in deeper layers of the target tissue. Two of them are based on the use of chemicals, given along with ALA, in order to enhance both its penetration into deeper tissue layers and/or the total PpIX accumulation. This transepithelial penetration enhancement can be achieved either by prior dimethyl sulphoxide exposure of the targeted area (Peng et al, 1995) or by encapsulation of ALA into liposomes (Fukuda et al, 1992). The second approach uses agents interfering directly with the biosynthetic pathway of haem. Tetrapyrrol modulators, such as 1,10-phenanthroline (Rebeiz et al, 1996) and allyl-isopropyl-acetamide (AIA) (Schoenfeld et al, 1994) stimulating the enzymatic activity associated with PpIX formation. Iron chelators (e.g. ethylenediaminotetraacetic acid (EDTA) (Hanania and Malik, 1992; Orenstein et al, 1995; Warloe et al, 1995), desferrioxamine (DFO) (Ortel et al, 1993) CP94 (Chang et al, 1997) have been shown to increase PpIX concentration by preventing the ferrochelatase-mediated insertion of iron into the tetrapyrrol ring. This study followed a third approach, based on the thesis that the transformation of the hydrophilic ALA into more lipophilic prodrugs will enhance drug uptake.

In view of the results obtained using esters of ALA in vitro (Kloek et al, 1996; Gaullier et al, 1997; Marti et al, 1999; Tyrrell et al, 1993) and in vivo (Kloek et al, 1996; Peng et al, 1996), it appeared reasonable to envisage developing such a substance for clinical tests in which superficial bladder carcinoma is detected by fluorescence and possibly even treated by PDT. From the variety of derivatives recently tested in our laboratory (Marti et al, 1999), we selected h-ALA as it represents a good compromise between water–urine solubility and lipophilicity. It also gave an excellent in-vitro dose drug response compared to ALA solutions. Furthermore, it can be synthesized with a fairly high yield and low cost. The goal of this first clinical study with h-ALA in urology was to evaluate the feasibility of fluorescence photodetection with this new agent and the advantages achieved by instillation of h-ALA as compared to ALA for use in the human bladder.

One criterion for the use of h-ALA as a potential candidate in replacing ALA, is the preservation of the outstanding selectivity of ALA-induced PpIX for malignant and pre-malignant tissues. Confirmed by histopathological examination, we have demonstrated that the fluorescence of PpIX in the urothelium induced by intravesically administered h-ALA correlated significantly with neoplastic lesions and was suitable for the detection of papillary tumours as well as for dysplasia and carcinoma in situ. The 7% rate of false negative responses found in the present study is comparable to the value given by Jichlinski et al in 1997 and slightly higher than that presented by the Munich group (Kriegmair et al, 1996). A total number of 28 biopsies were taken from areas proven to be benign. Only five of these samples revealed an enhanced red fluorescence under violet light irradiation, yielding a rate of falsely positive fluorescence findings of 17%. This result seems to be quite small compared to both the results of Kriegmair et al (1996) and Jichlinski et al (1997). But it may be explained by the small number of biopsies taken, or the fact that the fluorescence induced by the long-chain esters was found to be limited to the site of application (Peng et al, 1996), hence no supplementary PpIX build-up from systemic ALA uptake is observed.

Clinical fluorescence spectroscopy has been used for measuring the PpIX accumulation kinetics, indicating an increase of h-ALA-induced PpIX with time in the human bladder within 6 h. A quantitative comparison of the fluorescence intensities at 636 nm following similar instillation conditions with solutions of 180 mM of ALA or 8 mM of h-ALA, respectively, clearly shows the advantages of h-ALA-induced PpIX. The more than twofold increase of the fluorescence signal due to the use of h-ALA is in good agreement with the in vivo results of Kloek et al (1996).

The time course of the PpIX fluorescence intensity in neoplastic tissues shows that, following 8 mM h-ALA exposure for 2 or 4 h, synthesis of PpIX continues within almost 2 h after termination of the instillation. In this time range, the fluorescence intensity increases 400% (2 h of exposure, 2 h of resting time) and 25% (4 h of exposure, 2 h of resting time) respectively.

The significant increase of the fluorescence signal using the ‘(2+2)-concept’ as compared to a permanent exposure to drug for 4 h, as well as the strong dependence on the instilled h-ALA concentration, can be explained by an interference of high ALA concentrations with the biosynthetic pathway of haem. This observation was confirmed by in vitro experiments made by Gaullier et al (1997) and Marti et al (1999) with several ALA esters including h-ALA. Whereas the transport of ALA across the lipid bilayer of cell membranes probably represents a bottleneck in the PpIX formation, the enhanced uptake of lipophilic h-ALA may saturate the intracellular PpIX biosynthesis. This saturation might cause a negative feedback to enzymatic activity. Furthermore, high intracellular ALA concentrations have been shown to be cytotoxic. A high cellular ALA content may induce the release of $Ca^{2+}$ from mitochondria, mitochondria swelling and uncouple respiration (Hermes-Lima, 1995). It can also cause ferritin iron release (Berg et al, 1996) or mediate the formation of 8-hydroxy-2′-deoxyguanosine in DNA (Fraga et al, 1994).

The results presented in this study have shown that a 2 h instillation of h-ALA (8 mM) provides sufficient PpIX fluorescence for reliable photodetection of malignant and pre-malignant lesions. This reduction in instillation time to only 2 h significantly increases the patient’s comfort. Moreover, this makes outpatient treatment feasible and helps to cut costs in view of the excessively increasing cost of hospitalization. Finally, the reduction of the drug dose will decrease drug cost and the potential risk of mild complications provoked by ALA, recently reported by Rick et al (1997).

While for reliable fluorescence photodetection a 2 h instillation of 8 mM h-ALA has been shown to give satisfactory results, other conditions must be fulfilled with respect to an efficient bladder cancer therapy by PDT. Among other factors, the two key parameters of high concentration of the photosensitizer and its homogeneous distribution in the target tissue play a major role for the effectiveness of PDT.

Fluorescence microscopic studies showed that, after topical application of ALA, the PpIX was restricted to the superficial layers of the bladder tumours (Steinbach et al, 1994). On the contrary, preliminary fluorescence microscopic studies on some biopsies, taken in this study (data not shown), as well as the in vitro studies of Marti et al (1999) demonstrated homogeneously distributed PpIX fluorescence over the entire urothelium after topical application of h-ALA solutions.

Considering the photobleaching of porphyrins during irradiation (Rotomski et al, 1996; Bezdetnaya et al, 1996; Moan et al, 1997), a threshold concentration of PpIX necessary for tissue destruction, and the high selectivity of h-ALA-induced PpIX, a small PpIX amount in healthy areas of the bladder, observed in this study will probably not induce any damage in these regions. However, the twofold increase of PpIX fluorescence after 6 h in neoplastic tissues by using h-ALA may further enhance the PDT effect as compared to the use of ALA. The latter appeared insufficient as observed in recent studies (Kriegmair et al, 1996).

It can be concluded that the use of h-ALA is a promising way to improve the photodetection of neoplastic and pre-neoplastic lesions as compared to ALA. In future, h-ALA may replace the use of ALA for clinical intravesical instillation because it is easy to use, real time observation without major auxiliary devices is possible and it is relatively cheap. Finally, it looks more promising as a PDT agent than ALA itself.

### ACKNOWLEDGEMENTS

The authors are grateful to the Swiss 'Fonds National', the Common Research Program in biomedical technology between Lausanne Hospital (CHUV), the Swiss Federal Institute of Technology (EPFL), Lausanne University (UNIL), the Swiss National Priority Program in Optics, and the 'Fonds Vaud-Geneva' for their financial support. Norbert Lange thanks Patrick Gerber (ICO, University of Lausanne) for many fruitful discussions. The Deutsche Forschungsgemeinschaft (DFG), Bonn, Germany, provided the grant for Dr N Lange.

### REFERENCES

Berg K, Anholt H, Bech O and Moan J (1996) The influence of iron chelators on the accumulation of protoporphyrin IX in 5-aminolaevulinic acid-treated cells! *Br J Cancer* **74**: 688–697

Bezdetnaya L, Zeghari N, Belitchenko I, Berberi-Heyob M, Merlin JL, Potapenko A and Guillemin F (1996) Spectroscopic and biological testing of photobleaching of porphyrins in solutions. *Photochem Photobiol* **64**: 382–386

Bridges JW, Sargent NSE and Upshall DG (1979) Rapid absorption from the urinary bladder of a series of n-alkyl carbamate: a route for the recirculation of drug. *Br J Pharmacol* **66**: 283–289

Chang SG, MacRobert AJ and Bown SG (1996) Biodistribution of protoporphyrin IX in rat urinary bladder after intravesical instillation of 5-aminolaevulinic acid. *J Urol* **155**: 1744–1748

Chang SG, MacRobert AJ, Porter JB and Bown SG (1997) The efficacy of an iron chelator (CP94) in increasing cellular protoporphyrin IX following 5-aminolaevulinic acid administration: an in vivo study. *J Photochem Photobiol B* **38**: 114–122

Cox GS, Bobillier C, Whitten DG (1982) Photo-oxidation and singlet oxygen sensitization by protoporphyrin IX and its photo-oxidation products. *Photochem Photobiol* **36**: 401–407

Dougherty TJ, Cooper MT and Mang TS (1990). Cutaneous phototoxic occurrences in patients receiving Photofrin. *Lasers Surg Med* **10**: 485–488

Fraga CG, Onuki J, Lucesoli F, Bechara EJ and Di Mascio P (1994) 5-Aminolaevulinic acid mediates the in vivo and in vitro formation of 8-hydroxy-2'-deoxyguanosine in DNA. *Carcinogenesis* **15**: 2241–2244

Fukuda H, Paredes S and Del Battle AM (1992) Tumor localizing properties of porphyrins in vivo studies using free and liposome encapsulated aminolaevulinic acid. *Comp Biochem Physiol* **102b**: 433–436

Gaullier JM, Berg K, Peng Q, Anholt H, Selbo PK, Ma LW and Moan J (1997) Use of 5-aminolaevulinic acid esters to improve photodynamic therapy on cells in culture. *Cancer Res* **57**: 1481–1486

Hanania J and Malik Z (1992) The effect of EDTA and serum on endogenous porphyrin accumulation and photodynamic sensitization of human leukemic cells. *Cancer Lett* **65**: 127–131

Hermanek P and Sobin J (1992) *UICC TNM Classification of Malignant Tumours*, 4th edn. Springer Verlag: Berlin

Hermes-Lima M (1995) How do $Ca^{2+}$ and 5-aminolaevulinic acid-derived oxyradicals promote injury to isolated mitochondria? *Free Radical Biol Med* **19**: 381–390

Iinuma S, Bachor R, Flotte T and Hasan T (1995). Biodistribution and phototoxicity of 5-aminolaevulinic acid-induced PpIX in an orthotopic rat bladder tumor model. *J Urol* **153**: 802–806

Jain RK (1987*a*) Transport of molecules in the tumor interstitium: a review. *Cancer Res* **47**: 3039–3305

Jain RK (1987*b*). Transport of molecules across tumor vasculature. *Cancer Metast Rev* **6**: 559–593

Jichlinski P, Forrer M, Mizeret J, Glanzmann T, Braichotte D, Wagniéres G, Zimmer G, Guillou L, Schmidlin FM, Graber P, van den Bergh H and Leisinger HJ (1997) Clinical evaluation of a method for detecting superficial transitional cell carcinoma of the bladder by light-induced fluorescence of protoporphyrin IX following topical application of 5-aminolaevulinic acid: preliminary results. *Lasers Surg Med* **20**: 402–408

Kennedy JC, Pottier RH and Pross DC (1990) Photodynamic therapy with endogenous protoporphyrin IX: basic principles and present clinical experience. *J Photochem Photobiol. B* **6**: 143–148

Kloek J and Beijersbergen van Henegouwen GMJ (1996) Prodrugs of 5-amino-laevulinic acid for photodynamic therapy. *Photochem Photobiol* **64**: 994–1000

Kriegmair A, Baumgartner R, Knuechel R, Steinbach P, Ehsan A, Lumper W, Hofstaeder W and Hofstetter A (1994) Fluorescence photodetection of neoplastic urothelial lesions following intravesical instillation of 5-aminolaevulinic acid. *Urology* **44**: 836–841

Kriegmair M, Baumgartner R, Lumper W, Waidelich R and Hofstetter A (1996) Early clinical experience with 5-aminolaevulinic acid for the photodynamic therapy of superficial cancer. *Br J Urol* **77**: 667–671

Leveckis J, Burn JL, Brown NJ and Reed MWR (1994) Kinetics of endogeneous protoporphyrin IX induction by aminolaevulinic acid: preliminary studies in the bladder. *J Urol* **152**: 550–553

Levi F (1993) Incidence of infiltrating cancer following superficial bladder carcinoma. *Int J Cancer* **55**: 419–421

Loh CS, Vernon D, MacRobert AJ, Bedwell J, Bown SG and Brown SB (1993) Endogenous porphyrin distribution induced by 5-aminolevulinic acid in tissue layers of the gastrointestinal tract. *Photochem Photobiol* **20**: 47–54

Marti A, Lange N, van den Bergh H, Sedmera D, Jichlinski P and Kuchera P (1998) Optimalisation of the formation and distribution of protoporphyrin IX in the urothelium: an in vitro approach. *J Urol* (in press)

Moan J, Streckyte G, Bagdonas S, Bech O and Berg K (1997) Photobleaching of protoporphyrin IX in cells incubated with 5-aminolevulinic acid. *Int J Cancer* **70**: 90–97

Mostofio FK, Sobin LH and Torloni H (1973) Histological typing of urinary bladder tumors. In *International Histological Classification of Tumors*. World Health Organization Geneva

Nagy GK, Frable WJ and Murphy WM (1982) Classification of premalignant urothelial abnormalities: A Delphi study of the National Bladder Cancer Collaborative Group A. In Sommers SC and Rosen PP (eds) pp. 219–233 Appleton: Norwalk, CT

Novo M, Huettmann G and Diddens H (1996) Chemical instability of 5-aminolaevulinic acid used in the fluorescence diagnosis of bladder tumours. *J Photochem Photobiol B* **34**: 143–148

Orenstein A, Kostenich G, Tsur H, Roitman L, Ehrenberg B and Malik Z (1995) Photodynamic therapy of human skin tumors using topical application of 5-aminolaevulinic acid, DMSO and EDTA. *Proc SPIE* **2325**: 100–105

Ortel B, Tanew A and Honigsmann H (1993) Lethal photosensitization by endogenous porphyrins of PAM cell-modification by desferrioxamine. *J Photochem Photobiol B* **17**: 273–278

Peng Q, Moan J, Warlow T, Nesland JM and Rimington C (1992) Distribution and photosensitizing efficiency of porphyrins induced by application of endogenous 5-aminolaevulinic acid in mice bearing mammary carcinoma. *Int J Cancer* **52**: 433–443

Peng Q, Warloe T, Moan J, Heyerdahl H, Steen HB, Nesland JM and Giercksky KE (1995) Distribution of 5-aminolaevulinic acid-induced porphyrins in noduloulcerative basal cell carcinoma. *Photochem Photobiol* **62**: 906–913

Peng Q, Moan J, Warloe T, Irani V, Steen HB, Bjorseth A and Nesland JM (1996) Build-up of esterified aminolaevulinic-acid-derivative-induced porphyrin fluorescence in normal mouse skin. *J Photochem Photobiol B* **34**: 96–96

Rebeiz N, Arkins S, Rebebeiz CA, Simon J, Zachary JF and Kelly KW (1996) Induction of tumour necrosis by δ-aminolaevulinic acid and 1,10-phenanthroline photodynamic therapy. *Cancer Res* **56**: 339–344

Richie JP, Shipley WU and Yagoda A (1989) Cancer of the bladder. In *Cancer Principles and Practice of Oncology*, De Vita VT, Hellmann S and Rosenberg SA (eds), pp. 1008–1020. JB Lippincott: Philadelphia

Rick K, Sroka R, Stepp H, Kriegmair M, Huber RM and Baumgartner R (1997) Pharmacokinetics of 5-aminolaevulinic acid-induced protoporphyrin IX in skin and blood. *J Photochem Photobiol B* **40**: 319–313

Rotomski R, Bagdonas S and Streckyte G (1996) Spectroscopic studies of photobleaching and photoproduct formation of porphyrins used in tumour therapy. *J Photochem Photobiol B* **33**: 61–67

 © Cancer Research Campaign 1999

Schoenfeld N, Mamet R, Norenberg Y, Shafran M, Babuskin T and Malik Z (1994) Protoporphyrin biosynthesis in melanoma B 16 cells stimulated by 5-aminolaevulinic acid and chemical inducers: characterization of photodynamic inactivation. *Int J Cancer* **56**: 106–112

Steinbach P, Kriegmair M, Baumgartner R, Hofstadter F and Knuechel R (1994) Intravesical instillation of 5-aminolaevulinic acid: the fluorescent metabolite is limited to urothelial cells. *Urology* **44**: 676–681

Svanberg K, Andersson T, Killander D, Wang I, Stenram U, Andersson-Engels S, Berg R, Johansson J and Svanberg S (1994) Photodynamic therapy of non-melanoma malignant tumors of the skin using topical d-aminolaevulinic acid sensitization and laser irradiation. *Br J Dermatol* **130**: 743–751

Takeya H (1992) Preparation of 5-aminolaevulinic acid alkyl esters as herbicides. *Chem Abstr* **116**: P189633

Tyrrell RM, Pourzand C and van den Bergh H (1993) Unpublished results

Wagnière S, Hadjur C, Grosjean P, Braichotte D, Savary JF, Monnier P and van den Bergh H (1998) Clinical evaluation of cutaneous phototoxicity of 5, 10, 15, 20-tetra (m-hydroxyphenyl) chlorin. *Photochem Photobiol* **68**: 382–387

Warloe T, Peng Q, Steen HB and Gierchsky KE (1992) Localization of porphyrins in human basal cell carcinoma and normal tissue induced by topical application of 5-aminolaevulinic acid. In *Photodynamic Therapy and Biomedical Lasers*, Spinelli P, Dal Fante M and Marchesini R (eds), pp. 454–458. Elvesier Science: Amsterdam

Warloe T, Peng Q, Heyerdahl H, Moan J, Stenn HB and Giercksky KE (1995) Photodynamic therapy with 5-aminolaevulinic acid induced porphyrins and DMSO/EDTA for basal cell carcinoma. *Proc SPIE* **2371**: 226–235

*© Cancer Research Campaign 1999*
